# Glutamate acts as a key neurotransmitter for itch in the mammalian spinal cord

**DOI:** 10.1177/17448069231152101

**Published:** 2023-01-16

**Authors:** Qi-Yu Chen, Min Zhuo

**Affiliations:** 1 Qingdao International Academician Park, International Institute for Brain Research, Qingdao, China; 2 CAS Key Laboratory of Brain Connectome and Manipulation, Interdisciplinary Center for Brain Information, The Brain Cognition and Brain Disease Institute, Shenzhen-Hong Kong Institute of Brain Science-Shenzhen Fundamental Research Institutions, Shenzhen Institute of Advanced Technology, Chinese Academy of Sciences Shenzhen Institute of Advanced Technology, Shenzhen, China; 3 Department of Physiology, Faculty of Medicine, 7938 University of Toronto, Toronto, ON, Canada

**Keywords:** Itch, gastrin-releasing peptide, glutamate, anterior cingulate cortex

## Abstract

Itch sensation is one of the major sensory experiences of humans and animals. Recent studies using genetic deletion techniques have proposed that gastrin-releasing peptide (GRP) is a key neurotransmitter for itch in the spinal cord. However, these studies are mainly based on behavioral responses and lack direct electrophysiological evidence that GRP indeed mediates itch information between primary afferent fibers and spinal dorsal horn neurons. In this review, we reviewed recent studies using different experimental approaches and proposed that glutamate but not GRP acts as the key neurotransmitter in the primary afferents in the transmission of itch. GRP is more likely to serve as an itch-related neuromodulator. In the cerebral cortex, we propose that the anterior cingulate cortex (ACC) plays a significant role in both itch and pain sensations. Only behavioral measurement of itch (scratching) is not sufficient for itch measurement, since scratching the itching area also produces pleasure. Integrative experimental approaches as well as better behavioral scoring models are needed to help to understand the neuronal mechanism of itch and aid future treatment for patients with pruritic diseases.

## Introduction

Itch sensation is one of the major sensory experiences of humans and animals. Acute itch alerts an organism to harmful external threats, whereas chronic itch is a debilitating disorder that can cause skin excoriations and lead to sleep deprivation, anxiety, and depression.^1^ Itch induced by chemical or mechanical stimuli is transmitted by peripheral pruriceptive neurons to the spinal cord dorsal horn, then it is carried to the thalamus and further to cortices. Recent findings have suggested several candidate neurotransmitters that may mediate the transmission of pruritic information in the spinal cord dorsal horn. Glutamate and a few neuropeptides such as gastrin-releasing peptide (GRP) and natriuretic peptide B (NPPB) are promising candidates for an itch-specific peptide transmitter in primary neurons.^2–4^ As a neurotransmitter, one needs to meet at least the following criteria: (1) It is synthesized in the presynaptic neuron. (2) It is present in the presynaptic terminal and is released in amounts sufficient to exert a defined action on the postsynaptic neuron or effector organ. (3) When administered exogenously in reasonable concentrations it mimics the action of the endogenous transmitter (for example, it activates the same ion channels or second-messenger pathway in the postsynaptic cell). (4) A specific mechanism usually exists for removing the substance from the synaptic cleft.^5^ In addition to GRP, neuropeptides such as NPPB and neuromedin B (NMB), are also involved in peripheral itch transmission. Although the GRP-GRPR, NMB-NMBR, and NPPB-NPRA/NPRC signaling pathways have been proven to encode itch information in a non-complimentary manner,^6,7^ their presynaptic origins and postsynaptic receptors have been detected individually. However, it is lacking sufficient evidence for GRP, NMB, and NPPB to meet criteria 3 and 4.

## Early studies of itching

Chemical agents that elicit itch in humans, as well as animals, have been used as pruritogens in experimental studies. Histamine and other non-histaminergic agents such as cowhage, bovine adrenal medulla 8–22 peptide (BAM8-22), and chloroquine are commonly-used to induce itch and peripheral pruriceptor can be divided by histamine and non-histamine pruriceptors.^7–9^ It’s worth noting that pruriceptive neurons are subsets of a larger population of neurons that respond to noxious stimuli.^2^ The itch induced by each pruritogen was typically accompanied by slightly weaker and shorter-lasting nociceptive sensations. Therefore, peripheral pruriceptive neurons can be further classified according to their responsiveness to noxious mechanical, thermal, or chemical stimuli.

Recording from C-fibers in monkeys and humans and from dorsal root ganglion (DRG) neurons in mice evoked by histamine demonstrated that, the neuronal responses of C-fiber in humans,^10^ mice,^11^ and monkeys^12^ match the time course of itch in humans,^2^ suggesting that histamine activates pruriceptors with unmyelinated C-fiber to elicit itch.

## Gastrin-releasing peptide and itch: Is gastrin-releasing peptide a selective transmitter for itch?

Gastrin-releasing peptide (GRP) is the ligand for GRPR and was first found to be a mammalian homologue of the amphibian neuropeptide bombesin,^13^ and this peptide is evolutionarily conserved in vertebrates. Although its original roles discovered in the central nervous system are not related to itching, some studies combining in situ hybridization, reverse transcription-PCR, western blot, and immunohistochemical staining reported GRP and its mRNA have been detected in DRG neurons in various animal species, including rodents, dogs, monkeys and humans.^3,7,14–16^ Therefore, a few investigators believed that these GRP- expressing DRG neurons are the primary afferent origin GRP-positive neurons in the mouse dorsal horn.^17,18^ Furthermore, the GRP was considered as the neuropeptide code for non-histaminergic itch,^7,19^ conditional knockout of Grp in sensory neurons results in attenuated non-histaminergic itch, without impairing histamine-induced itch.^20^ However, the relationship between the location of the expression of GRP and the role of GRP still requires further investigation and discussion.

Cumulative studies have proved that Grp mRNA is abundantly expressed in lamina II of the mammalian dorsal horn, and GRP- immunoreactive fibers have been detected in the dorsal horn of the mammalian spinal cord.^3,4^ In the spinal dorsal horn, GRP and its receptor GRPR play a central role in itch transmission.^21–23^ Provoking itch-like behavior activation of spinal GRP neurons required mimicking the endogenous firing of GRP neurons. Mice lacking the GRPR exhibit strongly reduced responses to histamine-dependent and histamine-independent pruritogens.^3^ Local spinal ablation of the neurons that express the GRPR almost completely blocked itching evoked by a broad variety of pruritogens.^4^ Recent studies suggests that dorsal horn interneurons that are activated by peripheral pruriceptive neurons release GRP and in turn excite other GRPR-expressing interneurons that finally transmit pruriceptive signals to spinoparabrachial projection neurons.^16,24,25^ Immunochemical evidence has been provided that spinal GRPR-expressing neurons were heterogeneous. Only 62% of them co-expressed Lmx1b, while 27% expressed the inhibitory marker Pax2. In situ hybridization analyses of the vesicular glutamate transporter subtype 2 (Vglut2) and the vesicular GABA transporter subtype (VGAT) revealed that 81% of GRPR-expressing neurons were excitatory and 19% inhibitory neurons.^21^ There is research reported that GRPR-expressing cells in the spinal cord are predominantly excitatory interneurons that are found in the dorsal lamina, especially in laminae II–IV.^23^ An early study also reported that bombesin selectively depressed superficial dorsal horn neurons in laminae I-III, which was independent of the inhibitory system mediated by opioids, GABA or purines.^26^ This mechanism is different from how it works in cortices, the anterior cingulate cortex (ACC) for instance. In the ACC, GRP activates the inhibitory interneurons and enhanced spontaneous GABAergic, but not glutamatergic neurotransmission.^27^ Although GRP is one of the neuropeptides mediating spinal pruriceptive transmission, its role in primary afferents transmitting itching information from afferent fibers to spinal dorsal horn is still controversial. There is no direct evidence is available to indicate that GRP mediates between afferent fibers and dorsal horn neurons.

## Glutamate is the major neurotransmitter in the primary afferents

**Figure 1.**
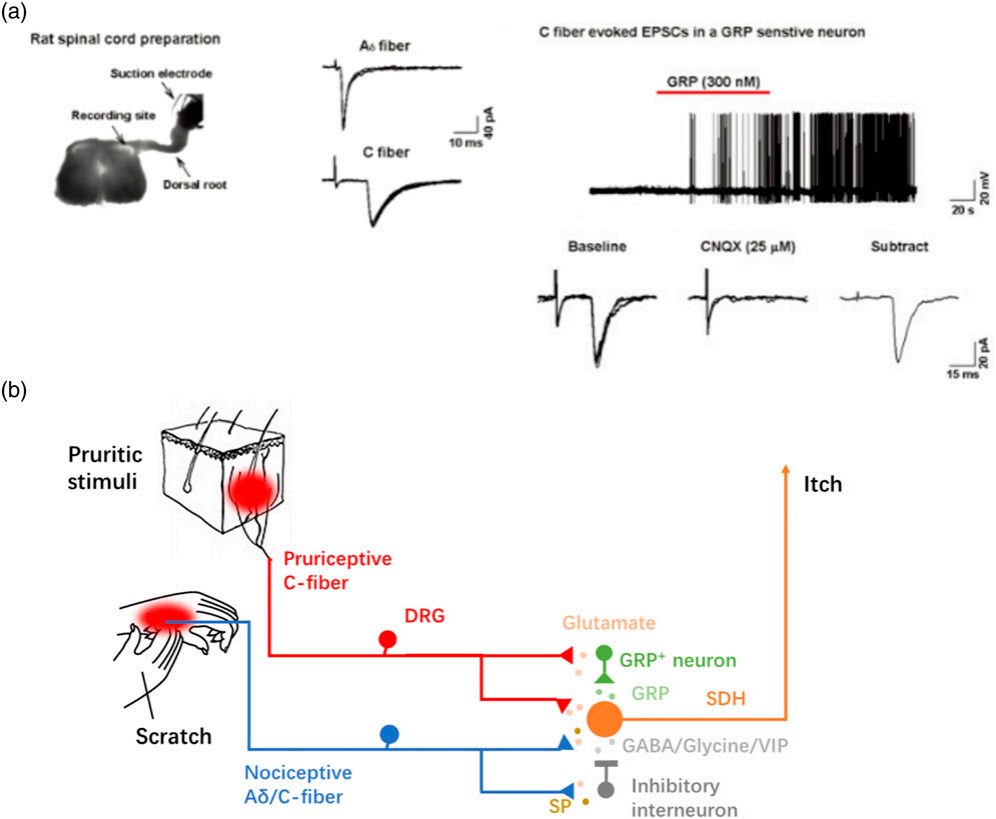
Glutamate instead of GRP is the major neurotransmitter in primary afferent fibers in itch transmission (a) (Left) Digitized photomicrograph showing one example of whole-cell patch recording on neurons in the superficial lamina of the spinal cord, which was stimulated by dorsal roots. (Middle) Examples of Aδ and C fiber evoked monosynaptic EPSCs. (Right) In a GRP-sensitive neuron, a monosynaptic C fiber-evoked EPSC was blocked by a bath application of CNQX (25 μM), an AMPA/KA receptor antagonist. (b) Itch and scratch stimuli activate dorsal root ganglion (DRG) neurons which synapse in the dorsal horn of the spinal cord. Pruritogen-responsive primary afferent fibers project directly or indirectly onto neurons in the spinal dorsal horn (SDH) and make another synapse onto GRP^+^ interneurons. The major neurotransmitter for primary afferent fibers is glutamate. Activation of a nociceptive A_δ_/C fiber that projects onto inhibitory interneurons is proposed to have strong inputs to the SDH neuron and can block the response evoked by pruriceptive stimuli.

In one of our previous studies, by performing in vitro spinal afferent stimulation and whole-cell patch-clamp recording, Koga et al. found that a part of superficial dorsal horn neurons responded to GRP application with the increase of action potential firing in adult rats and mice, and these dorsal horn neurons received exclusively primary afferent C-fiber inputs^28^ (Figure 1). On the other hand, few Aδ inputs receiving cells were found to be GRP positive. CNQX, a blocker of AMPA and kainate (KA) receptors, completely inhibited evoked EPSCs, including in those Fos-GFP positive dorsal horn cells activated by itching. These results provide direct evidence that glutamate, but not GRP, is the principal excitatory transmitter between C fibers and GRP-positive dorsal horn neurons.

In addition to GRP, a family of G protein-coupled receptors, Mas-related G protein-coupled receptors (MRGPRs), encode receptors for itch-inducing substances, for example, chloroquine.^29^ In primary afferents expressing MRGPRA3, which highly expresses Vglut2 and the NMB, optogenetic stimulation of MRGPRA3^+^ afferents triggers scratching and other itch-related avoidance behaviors. Results combining optogenetics and spinal cord slice recording showed that glutamate is essential for MRGPRA3^+^ afferents to transmit itch.^30^ This is in contrast to those reported that glutamate is required for nociception but dispensable for itch transmission.^31^ Genetic ablation of Vglut2 in primary nociceptors and itch-sensing afferents leads to decreased pain but increased itch behaviors.^32,33^ However, it is worth noting that no physiological recordings have directly confirmed that glutamate transmission is disrupted in the primary itch-sensing afferents of Vglut2 conditional knockout mice.

## Other roles of gastrin-releasing peptide in the central nervous system

Gastrin-releasing peptide (GRP) is the mammalian homolog of the amphibian 14-amino acid peptide bombesin, isolated from the skin of the European frog *Bombina bombina* in 1970. In the late 1980s, Flood and Morley (1988) demonstrated that systemic or i.c.v. injections of GRP facilitated memory consolidation after learning modulated memory retention whereas systemic injections produced memory enhancement or impairment depending on the drug dose and training conditions.^34^ Pre- or post-training injection of GRPR selective antagonists impaired the inhibitory avoidance memory.^35,36^ In mice lacking GRPR, contextual and cued fear conditioning were enhanced, whereas spatial memory in the Morris water maze was unaffected. The enhancement of fear memory in GRPR knockout mice was accompanied by long-term potentiation (LTP) recorded in the lateral nucleus of the amygdala, in which GRPR is expressed in GABAergic interneurons of the lateral nucleus. GRP excites these interneurons and increases their inhibition of principal neurons.^37^ Recent in vivo photometry and CRISPR-Cas9-mediated knockout of the GRPR in the auditory cortex indicate that VIP cells are strongly recruited by novel sounds and aversive shocks, and GRP-GRPR signaling enhances auditory fear memories.^38^ GRP and GRPR can be activated by stressful stimuli, thus GRPR signaling is likely to be a major regulator of memory associated with fear and emotional arousal. GRP may stimulate the release of adrenocorticotropic hormone, playing a role in mediating the corticotropin-releasing hormone stress response, and increasing the activity of the hypothalamic-pituitary-adrenal axis.^39^ Systemic administration of a GRPR antagonist can induce an anxiogenic-like effect in the elevated plus maze test in rats.^40^ In addition, GRP-GRPR systems have also been reported to play roles in feeding and sex behaviors by interacting with the central nervous system.^41,42^

## Itch and pain transmission pathways

**Figure 2.**
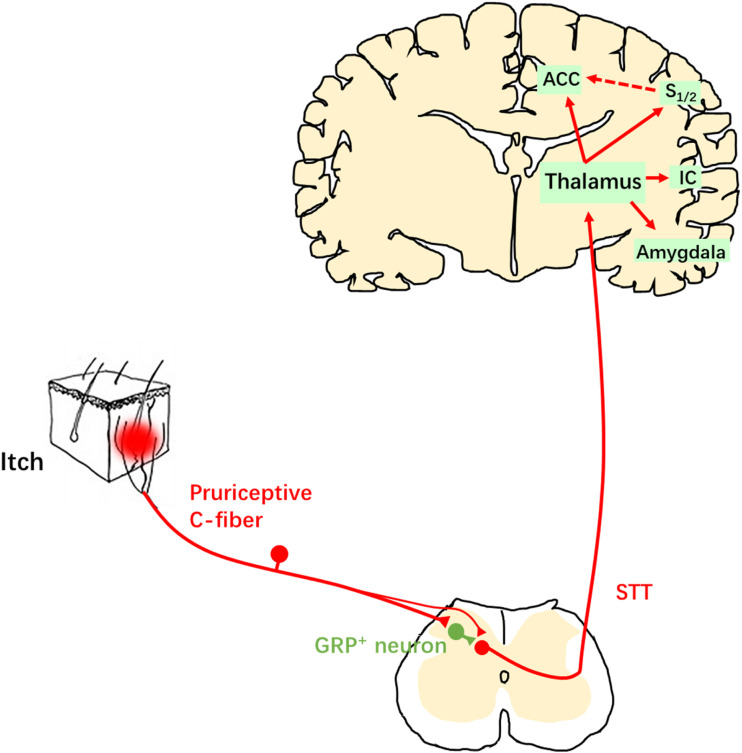
Itch transmission pathway Pruritic information ascends in the spinothalamic tract (STT) to the thalamus. Thalamus sends projections to the cortices which are involved in pruriceptive transmission, for example, the ACC, IC, and primary/secondary somatosensory cortex (S1/2). The cortical-cortical projection, for example, the projection from the S1/2 to the ACC, may be involved in the affective component of the itch sensation.

As we mentioned above, there is no evidence for the existence of itch-specific peripheral sensory neurons that is responsive only to pruritic but not noxious stimuli. However, non-pruriceptive nociceptive neurons have been identified that are unresponsive to pruritic chemicals.^2^ Peripheral pruriceptive nociceptive primary sensory neurons as well as non-pruriceptive nociceptive neurons terminate in the spinal dorsal horn, where they project to interneurons and spinothalamic tract (STT) neurons (Figure 2). The axons of STT neurons and other projection neurons from the spinal cord ascend within the anterolateral funiculus. Transection of this ascending pathway impairs itch as well as pain and temperature sensations. Pruriceptive information is transmitted from the thalamus to brain cortices that are involved in itch sensation, for example, the somatosensory cortex, ACC and insular cortex (IC).^43–46^

## Different coding theories of itch

Similar to pain, an itch sensation is formed in the brain. In the peripheral, many theories have been proposed to explain how itch is encoded and distinguished from pain. First, the specificity (or labeled line) theory indicates that itch- and pain-inducing stimuli are conveyed via distinct neural pathways from the peripheral to the spinal cord.^2,7,47^ According to this theory, there are itch-specific neurons in the periphery, which are activated by itch stimuli exclusively, but peripheral neurons with receptors for specific chemical pruritogens also respond to noxious stimuli that evoke pain. On the contrary, in wildtype mice, noxious stimuli such as a capsaicin injection, or noxious heat or mechanical stimuli, typically elicit pain rather than itch.^48^ Sufficient activation of pain-mediating neurons prevents or masks the effects of simultaneous activity in pruriceptive neurons, resulting in pain without itch. Therefore, a ‘selectivity (or population) theory’ indicates whether an itch or pain sensation occurs depends on the relative activity in neurons modulating itch or pain. Painful stimuli activate both non-pruriceptive and pruriceptive nociceptors, most non-pruriceptive nociceptors can inhibit that of the small subset of pruriceptive nociceptors.

To further elucidate the population theory of how different populations of neurons are activated, the intensity theory was carried out. According to this theory, itch and pain are transmitted by the same fibers, but are distinguished by the different firing intensities that they induce: weak activation of nociceptors provokes itch, whereas strong activation of the same population of neurons evokes pain.^7,47^ The firing intensity can also be regarded as the temporal pattern of activation. The detailed criteria of the firing intensity to distinguish itch from pain have not been established.

Another coding theory of itch is spatial theory. Since the distribution of pruriceptors may be different in the epidermis and dermis, when the itch-inducing stimuli are applied at a different location, a mixture of pruriceptors and nociceptors will be differentially activated, leading to different population firing patterns and thereby encoding itch or pain specificity.^49^ In spatial concerns, itch may occur when there is a ‘spatial contrast’ between the activity of one or a few pruriceptors and the absence of activity in neighboring nociceptors.^50,51^

These coding theories focused primarily on the coding of itch versus pain by the primary sensory neurons. They also demonstrated that itch and pain information is distinctly relayed to the spinal cord. The difference in the ascending pathway and upper brain areas remains to be investigated.

## The potential role of cortical areas in itch

**Figure 3.**
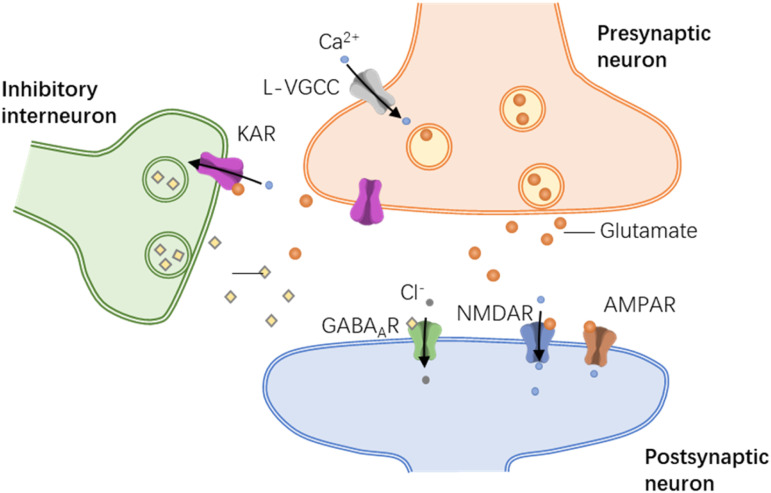
Pruritogen-dependent KA receptor modulation of evoked GABAergic transmission in the ACC. A model of ACC synaptic modulation during pruritogen-induced scratching. Peripheral application of itching stimuli activates excitatory afferents projecting to ACC layer II/III pyramidal neurons. Activity-dependent pre-synaptic glutamate release activates postsynaptic AMPA receptors located on pyramidal neurons thereby enhancing the post-synaptic response. Simultaneously, pre-synaptic glutamate can activate pre-synaptic kainate receptors (KAR) located on inhibitory neurons, thereby modulating GABA release and affecting the attenuation of the post-synaptic response.

In the forebrain, a few cortices are involved in itch processing. Imaging studies in humans indicate that many brain regions were found to respond to histamine and cowhage-induced itch such as the prefrontal cortex (PFC), motor cortex, the primary and secondary somatosensory cortex (S1/2), parietal cortex, cingulate cortex, and IC.^52^ Among them, the ACC plays a critical role in itch sensation.^53,54^ Rodent studies demonstrate that itch stimulation can activate neurons in the ACC.^46,55^ In the ACC, GluK1- containing kainate (KA) receptors are involved in scratching induced by histamine and non-histamine-dependent itching stimuli. Besides, scratching corresponds with the enhanced excitatory transmission in the ACC through KA receptor modulation of inhibitory circuitry (Figure 3).^46^ Activation of locus coeruleus noradrenaline projection to the ACC by optogenetic method induced scratching and behavioral sensitization for mechanical stimulation.^45^ The cortical network processing itch information ascended from the spinal cord and executing scratching behavior remains to be investigated.

## Scratching cannot be used as the only sign of itch: scratching triggers pleasure!

Scratching is often presumed to provide mechanical protection and subsequent inflammatory defense against harmful elements on the skin. However, scratching is also known to disrupt the epidermal barrier and facilitate infection.^56^ Another view is that we scratch because we want to relieve the itch by causing localized pain that will suppress the intolerable itch, suggesting we prefer to withstand mild pain rather than be itchy. Moreover, relieving an itch via scratching often causes a feeling of pleasure, thought to be due to both the riddance of the intolerable itch and the release of serotonin during scratching.^57^ Behavioral scratching scores are mostly used for measuring itch.^2,58^ However, scratching produces tactile and nociceptive sensory stimulation that do influence the sensations that the researcher wishes to measure. These site-directed responses are therefore indirect indicators of sensation.

Scratching activates spinal interneurons that inhibit itch-sensitive neurons, suppressing the transmission of itch signals to the brain.^59,60^ Investigation of human brain image found that the amygdala is deactivated by scratching.^61,62^ Since the amygdala mediates emotional disorders, thus negative emotions can be relieved by scratching. Independent from its role in itch relief, scratching is also pleasurable.

Specifically, scratching an itch is pleasurable in a way that scratching without itch does not produce pleasure, and actively scratching oneself is far more pleasant than being passively scratched by another person.^63^ The pleasure of scratching is correlated with activity in major reward circuits in the brain, such as the ventral tegmental area (VTA) and the nucleus accumbent. In the VTA, itch-induced aversion and scratch-induced pleasure are encoded by GABAergic and dopaminergic neurons individually, while the scratch-induced pleasure still depends on the itch.^64^ Pleasantness evoked by scratching activated not only the reward system but also key regions of perception as well as motor-related regions. This activation could explain why scratching-induced pleasantness potentially reinforces scratching behaviors.^63^ Therefore, we suggest to combine the electrophysiological evidences that itching instead of scratching activate specific neurons or pathways with the scratching behavior when investigating the itch sensation.

## Anterior cingulate cortex modulates pleasure after scratching

Anterior cingulate cortex (ACC) can be activated by pleasurable events.^65–68^ Interestingly, one brain imaging work reported that the ACC is significantly deactivated after active or passive scratching of itch.^62^ Regression analyses of brain activity versus pleasurability ratings showed that areas including the ACC deactivated while actively scratching an itch coincided with areas significantly correlated with pleasurability. Our previous study also proved that the ACC is involved in modulating pleasure.^69^ The detailed pattern of how the ACC modulates scratching-induced pleasure requires future investigation.

## Conclusion and future directions

In conclusion, glutamate acts as the central neurotransmitter in primary afferents of the spinal cord dorsal horn, while GRP and other neuropeptides modulate itch transmission. The itching sensation is primarily formed in the cortices, especially the ACC. The detailed central transmission pathway and key molecules require further investigation.
